# Analysis of polymorphisms in the circadian-related genes and breast cancer risk in Norwegian nurses working night shifts

**DOI:** 10.1186/bcr3445

**Published:** 2013-07-03

**Authors:** Shanbeh Zienolddiny, Aage Haugen, Jenny-Anne Sigstad Lie, Helge Kjuus, Kristine Haugen Anmarkrud, Kristina Kjærheim

**Affiliations:** 1 Department of Chemical and Biological Work Environment, National Institute of Occupational Health, PB 8149 Department, N-0033 Oslo, Norway; 2 Department of Occupational Medicine and Epidemiology, National Institute of Occupational Health, Oslo, Norway; 3 Cancer Registry of Norway, Institute of Population-based Cancer Research, Oslo, Norway

**Keywords:** Breast cancer, Circadian, Clock, Night shift, SNP

## Abstract

**Introduction:**

Some studies have suggested that night work may be associated with an increased risk of breast cancer in nurses. We aimed to explore the role of circadian gene polymorphisms in the susceptibility to night work-related breast cancer risk.

**Methods:**

We conducted a nested case-control study of Norwegian nurses comprising 563 breast cancer cases and 619 controls within a cohort of 49,402 Norwegian nurses ages 35 to 74 years. We studied 60 single-nucleotide polymorphisms (SNPs) in 17 genes involved in the regulation of the circadian rhythm in cases and controls. The data were analyzed in relation to the two exposure variables "maximum number of consecutive night shifts ever worked" and "maximum number of consecutive night shifts worked for at least 5 years." The odds of breast cancer associated with each SNP was calculated in the main effects analysis and in relation to night shift work. The statistically significant odds ratios were tested for noteworthiness using two Bayesian tests: false positive report probability (FPRP) and Bayesian false discovery probability (BFDP).

**Results:**

In the main effects analysis, CC carriers of rs4238989 and GG carriers of rs3760138 in the *AANAT *gene had increased risk of breast cancer, whereas TT carriers of *BMAL1 *rs2278749 and TT carriers of *CLOCK *rs3749474 had reduced risk. The associations were found to be noteworthy using both the FPRP and BFDP tests. With regard to the effect of polymorphisms and night work, several significant associations were observed. After applying FPRP and BFDP in women with at least four night shifts, an increased risk of breast cancer was associated with variant alleles of SNPs in the genes *AANAT *(rs3760138, rs4238989), *BMAL1 *(rs2290035, rs2278749, rs969485) and *ROR-b *(rs3750420). In women with three consecutive night shifts, a reduced risk of breast cancer was associated with carriage of variant alleles of SNPs in *CLOCK *(rs3749474), *BMAL1 *(rs2278749), *BMAL2 *(rs2306074), *CSNK1E *(rs5757037), *NPAS2 *(rs17024926), *ROR-b *(rs3903529, rs3750420), *MTNR1A *(rs131113549) and *PER3 *(rs1012477).

**Conclusions:**

Significant and noteworthy associations between several polymorphisms in circadian genes, night work and breast cancer risk were found among nurses who had worked at least three consecutive night shifts.

## Introduction

Breast cancer in women is the second leading cause of mortality [1]. The etiology of breast cancer involves several risk factors, such as early age at menarche, late age at menopause, late age at first birth, nulliparity, hormonal factors, ionizing radiation exposure and alcohol consumption [2,3]. However, these known risk factors do not fully explain the high incidence of breast cancer. Genetic factors are also involved in the etiology of breast cancer, including rare high-risk mutations (*BRCA1 *and *BRCA2*), more moderate susceptibility variants (*CHEK2*, *ATM*) and several as yet unidentified common susceptibility variants associated with low to moderate increased risk [4,5].

At present, there is not sufficient evidence for any occupational exposure to be classified as carcinogenic in relation to breast cancer. However, night work is a potential risk factor that has been found in many studies to be associated with an elevated risk of breast cancer [6-12]. A recent study reported that 4.6% of all female breast cancers could be attributed to occupational exposure to shift work [13]. This is important, given the large proportion of women engaged in shift work and the very high incidence of female breast cancer in general. Although the majority of epidemiological studies are suggestive of a positive association, not all studies show a consistent association between night work and increased risk of breast cancer [14-16].

Night work was classified as a Group 2A carcinogen by the International Agency for Research on Cancer (IARC) in 2007 [17,18]. The hypothesis of circadian disruption by light at night (LAN) has received particular attention in this evaluation [19]. Disruption of the circadian rhythm has been hypothesized to increase cancer risk through its impact on the production of the circadian hormone melatonin [20,21]. A potential mechanism could be through suppression of the nocturnal production of melatonin, caused by exposure to LAN, which may also influence the patterns of sex hormone production and in turn influence breast cancer risk [22,23]. The levels of melatonin are regulated through its biosynthesis from the amino acid tryptophan, which is mediated by the arylalkylamine *N*-acetyltransferase (AANAT) protein or melatonin receptors MTNR1A (MT1) and MTNR1B (MT2) and possibly the retinoic acid-related orphan receptors ROR-a and ROR-b [24]. An antiproliferative effect of melatonin has also been shown in human breast cancer cell lines. In estrogen receptor-positive (ER^+^) human breast cancer cells, melatonin suppresses both ER mRNA expression and estrogen-induced transcriptional activity of the ER [25,26]. Melatonin also regulates the transcriptional activity of other enzymes involved in estrogen metabolism and the expression of circadian and circadian-targeted genes. Recently, melatonin was found to induce apoptosis [27] and to reduce the levels of oxidative stress [28,29]. Other mechanisms, such as impact of sleep disruption on human physiological processes, may also be involved [30], although not all the results of studies on the impact of sleep disruption on breast cancer risk have been consistent [31,32].

The circadian clock is regulated by several proteins, such as aryl hydrocarbon receptor nuclear translocator-likes (ARNTL or BMAL1, ARNTL2 or BMAL2) also known as Mop3, circadian locomotor output cycle kaput (CLOCK), neuronal PAS domain protein 2 (NPAS2), periods (PER1, PER2 and PER3), cryptochromes (CRY1 and CRY2), TIMELESS, ROR-a and ROR-b, that interact with each other in various transcriptional and translational feedback loops [33]. The main signal is the daily light-dark rotation, which is mediated by light-sensing opsin proteins such as opsin 4 (OPN4) [34].

The circadian genes may affect cancer susceptibility through effects on biological pathways that regulate DNA damage and repair, carcinogen metabolism and/or detoxification, cell growth and cell death [35]. It has been shown that expression of the circadian gene *PER1 *may be downregulated in tumors and tumor cell lines compared to normal cells [36]. The *PER2 *gene has been shown to induce apoptosis through upregulation of the tumor suppressor gene *TP53 *and the proapoptotic gene *Bax *[37]. A *PER2 *gene mutation (S662G) also leads to enhanced resistance to X-ray-induced apoptosis and increased RAS-mediated oncogenic transformation, and it affects tumorigenesis in mice [38].

To date, there are no genetic susceptibility biomarkers identifying women who are particularly susceptible to breast cancer related to exposure to night work. In particular, knowledge of how night work, together with common genetic variants in genes regulating the circadian rhythm, may affect breast cancer risk is limited [39]. The aim of this study was to analyze a number of polymorphisms in the core circadian genes and genes involved in melatonin biosynthesis and melatonin bioavailability as potential biomarkers of breast cancer risk in night workers.

## Methods

### Case patients and controls

The details of study design, data collection, recruitment of cases and controls have been described previously by Lie *et al*. [8,9]. Briefly (see Additional File 1, Figure S1), a nested case-control study of 1,182 women ages 35 to 74 years was performed within an established national cohort of 49,402 Norwegian nurses who graduated between 1914 and 1985. All of the women had been diagnosed with histologically confirmed invasive breast cancer between 1990 and 2007 (*n *= 1,132), were alive as of February 2009 (*n *= 943), consented to be interviewed (*n *= 706) and had an occupational history as a nurse of at least 1 year (*n *= 699). The controls were frequency-matched to cases by year of diagnosis and in 5-year age groups. They were all cancer-free at and prior to the year of diagnosis of the case, were alive as of February 2009 (*n *= 1,384), had consented to an interview (*n *= 900) and worked as nurses for minimum of 1 year (*n *= 895). Breast cancer cases were identified by linkage to the Cancer Registry of Norway by personal identification number given to all Norwegians alive since 1960. The Cancer Registry of Norway has virtually complete records of all cancer cases in the Norwegian population since January 1953 [40]. Both cases and controls gave their full informed consent that their information could be used and published for research purposes, given that their personal details would remain anonymous. The study was approved by the Regional Committee for Medical and Health Research Ethics, South-East region (S-08430a, 2008/10453).

### Assessment of night work

Full details on the exposure assessment were described in a recent study [9]. Briefly, different exposure metrics of night work were computed on the basis of work history. The term *night work *in this study includes working periods from rotating as well as permanent night schedules. A "night shift" was defined as a shift including work between 12 a.m. and 6 a.m. For each job, we assessed information gathered for the year of start and end, workplace (hospital, other institution and/or others), proportion of full job and work schedule (only day shifts, only night shifts or both day and night shifts). If the job included night shifts, we also asked about the number of night shifts per month, and number of consecutive night shifts. Two surrogate measures for night work were applied: maximum number of consecutive night shifts in all jobs (<3, 3 and ≥4) and maximum number of consecutive night shifts the nurse had worked during a minimum of 5 years (<3, 3 or ≥4).

We focused our analysis on the variable consecutive night shift because, in our previous analysis [9], the consecutive night shift variable that takes duration into account was the only variable found to be significantly associated with an increased risk of breast cancer. In that previous analysis, a positive trend was seen with an increasing number of consecutive nights, albeit that a significant association was observed only when comparing those with at least 5 years of work in a job including six or more consecutive night shifts with those who never worked nights.

### Collection of saliva and DNA extraction

DNA from 563 saliva samples taken from cases and 619 samples obtained from controls were isolated using the Oragene·DNA isolation kit (DNA Genotek Inc, Kanata, ON, Canada) as instructed by the manufacturer, with some modifications. Briefly, 1 ml of saliva was transferred to an Eppendorf tube, and reagents provided by the supplier were added. DNA was successfully extracted from all saliva samples that were received.

### Selection of polymorphisms and genotyping

The single-nucleotide polymorphism (SNP) selection strategy was based primarily on a candidate gene approach using information from a search of relevant studies published by 2009. We searched the literature for polymorphisms in the core circadian genes and melatonin biosynthesis and/or bioavailability that had been investigated for an association with cancer risk. Since there were few polymorphisms that had been investigated by 2009, we also included polymorphisms using one or more of the following criteria: assumed functionality (located in the regulatory regions, for example, 3'-UTR, 5'-UTR or amino acid change), cancer genetic marker of susceptibility (CGEMS) for breast cancer in epidemiological studies (candidate gene or genome-wide association studies (GWASs)), candidate tag SNP with *r*^2 ^≥ 0.80 and minor allele frequency (MAF) ≥ 5%, and location in the relevant circadian, melatonin and light signaling pathways. Because of the constraint of the project budget, and hence limitations on the number of polymorphisms that could be placed on the chip, 60 SNPs were successfully designed and genotyped (see Additional File 2, Tables S1 and S2). The genotyping success rate was similar in cases and controls: at least 0.80 in cases and 0.82 in controls. All SNPs except for one SNP in the *CRY2 *gene (rs12364060) and one SNP in the *MTNR1A *gene (rs2119882) were in Hardy-Weinberg equilibrium. Two previously reported SNPs, rs35670208 in the *ARNTL2/BMAL2 *gene and rs28936679 in the *AANAT *gene, respectively, were found to be nonpolymorphic in this population. Genotyping was performed using the iPLEX genotyping assay (Sequenom Inc, San Diego, CA, USA) at the Centre for Integrative Genetics (CIGENE) genotyping core facility at the University for Environment and Biosciences, Ås, Norway.

### Statistical methods

Pearson's χ^2 ^test was used to assess case-control differences in the frequency distribution of categorical variables, and a nonparametric Mann-Whitney test was used to assess differences in median age between cases and controls. On the basis of existing knowledge of factors associated with breast cancer, the following factors were regarded as potential confounders: age at time of diagnosis (as a continuous variable in 5-years age groups), period of diagnosis (1996 to 1999 and 2000 to 2007), parity (0, 1 or 2 or ≥3 children), family history of breast cancer in mother and/or sister (no or yes), use of hormone treatment during the 2 years before the reference year (no or premenopausal, or yes) and frequency of alcohol consumption at time of diagnosis (less than twice weekly or at least twice per week). These variables were included in the final logistic regression model to obtain adjusted odds ratios (ORs) for developing breast cancer associated with the frequency of polymorphisms alone (main effects analysis), the polymorphisms and night work (stratified by number of consecutive night shifts). On the basis of the results of our previous study showing associations between intensity of night work and breast cancer risk, we examined the simultaneous effects of polymorphisms and night work by comparing the frequency of genotypes of each polymorphism in cases and controls who had worked either at least four and three consecutive night shifts with a reference group who had worked only day shifts or a maximum of two consecutive night shifts. We compared the frequency of SNPs in subgroups defined by both intensity (number of consecutive nights) and duration (number of years) of night work. In our previous study [9], the consecutive night shift variable that takes duration into account was the only variable found to be significantly associated with an increased risk of breast cancer, that is, a significant association was observed only when individuals with at least 5 years of work experience in a job including six or more consecutive night shifts were compared with those who had never worked nights. A positive trend was seen with increasing number of consecutive nights, however, thus, to avoid small numbers of subjects in the stratified analyses in the present study, women who had worked at least four consecutive night shifts were classified as the most exposed group. The relative risk of breast cancer associated with each genotype was estimated as OR with 95% confidence interval (CI) using unconditional logistic regression models. For determination of interactions between the shift work and SNP genotypes, the product of shift work × genotype variables was added to the model. Interaction *P *values (*P *interaction) are reported for the joint effects of shift work and genotype. All reported *P *values are two-sided. *P *< 0.05 was considered statistically significant. All statistical analyses were carried out using SPSS release 21 software (SPSSInc, Chicago, IL, USA).

### Multiple testing and false discovery rates

To evaluate the possibility of false-positives due to multiple testing, we applied two Bayesian statistical tests, false positive report probability (FPRP) and the Bayesian false discovery probability (BFDP), that have been used frequently to test noteworthiness of findings in genetic association studies [41-43]. The FPRP statistical tool was introduced by Wacholder *et al*. [42] as a means to assess whether the strength of an association is noteworthy in the case of multiple hypothesis testing. In addition, we used the BFDP, which is a recent further development of FPRP [41]. We performed both FPRP and BFDP tests for each SNP. For both tests, we chose the noteworthiness thresholds (FPRP 0.2 and BFDP 0.8) following the original papers [41,42]. We considered noteworthy only those associations that passed both tests. We have set low prior probability values compared to what commonly has been used [44].

## Results

The study design is a nested case-control study (see Additional File 1, Figure S1). The original cohort study of Norwegian nurses was described in detail by Lie *et al*. [8,9]. The demographic characteristics of cases and controls (Table 1) are not significantly different, except for the presence of breast cancer in mother or sister. The exposure parameters and the number of cases and controls in each exposure group are shown in Table 2. The selected circadian genes, chromosome locations and the protein function of each respective gene are shown in Additional File 2, Table S1. The SNPs with corresponding reference SNP (rs) numbers, SNP types, base changes, MAF, Hardy-Weinberg-derived *P *values and genotyping rates are given in Additional File 2, Table S2.

**Table 1:** Characteristics of controls and breast cancer cases.

Parameter	Cases (*n *= 563)	Controls (*n *= 619)	*P *value
Age (yr)^a^			0.51^b^
Mean (SD)	52.40 (7.88)	52.37 (8.18)	
Median	50	55	
Min/max	34/70	34/70	
Selection period			
1996 to 1999/2000 to 2007 (*n*)	195/368	221/398	0.72^c^
Breast cancer in first-degree family^d ^(*n*)	557	615	
Yes/no	104/453	54/561	<0.001^c^
Hormone therapy in past 2 yr^e ^(*n*)	552	605	
Yes/no	127/425	121/484	0.22^c^
Alcohol use^f^	*n *= 563	*n *= 619	
Yes/no	43/520	37/582	0.26^c^
Children (*n*)	563	619	
0/≥1	77/486	81/538	0.80^c^

^a^Age at selection for controls and age at diagnosis for cases. ^b^Derived from Mann-Whitney *U*-test for two independent samples (two-sided) assuming the medians are the same across cases and controls. ^c^Pearson's χ^2 ^test (two-sided). ^d ^Cancer in sister or mother. ^e^Hormone therapy among postmenopausal women only. ^f^Alcohol consumption at least twice weekly.

**Table 2:** Occupational exposure data and number of cases and controls in each exposure group.

Maximum number of consecutive night shifts in all jobs that nurse has worked^a^	No. of cases^b^	No. of controls^b^
Only day work or one or two consecutive night shifts (reference)	170 (227)	186 (275)
Three consecutive night shifts	156 (181)	189 (272)
Four or more consecutive night shifts	239 (291)	244 (348)
Total	563 (699)	619 (895)
**Intensity of night work: Maximum number of consecutive night shifts the nurse has ever worked for 5 or more years^c ^**		
Only day work or one or two consecutive nights for 5 or more years (reference)	326 (421)	362 (524)
Three consecutive night shifts for 5 or more years	125 (147)	154 (227)
Four or more consecutive night shifts for 5 or more years	112 (131)	103 (144)
Total	563 (699)	619 (895)

^a^Maximum number of consecutive night shifts in all jobs: 0 to 2, 3 or ≥4. ^b^Number of nurses who had consented to send saliva for genetic analysis (numbers in parentheses show the total number of cases and controls interviewed in each exposure group). ^c^Maximum number of consecutive night shifts the nurse has ever worked for 5 or more years.

Sixty SNPs in 17 genes (see Additional File 2, Tables S1 and Table S2) from the circadian signaling pathway were analyzed for association with breast cancer risk. As shown in Additional File 2, Table S2, the MAFs of the SNPs in controls were similar to those reported previously in Caucasian populations, indicating the validity of the genotyping methodology [45-47].

The analysis of main effects, without regard to night work (significant associations are shown in Table 3), showed that four SNPs were associated with breast cancer risk: CC carriers of the *AANAT *rs4238989 with an OR of 1.43 (95% CI, 1.01- 2.01) and GG carriers of *AANAT *rs3760138 with an OR of 1.42 (95% CI, 1.01- 2.01) had increased risk of breast cancer. The carriers of variant TT genotype of SNP rs2278749 in the *BMAL1 *gene with an OR of 0.45 (95% CI, 0.23- 0.90) and the homozygote genotype TT of rs3749474 in the *CLOCK *gene with an OR of 0.64 (95% CI, 0.45- 0.92) had reduced risk of breast cancer. All of these associations were found to be noteworthy for both FPRP (<0.2) and BFDP (<0.8).

**Table 3:** Odds ratios and 95% confidence intervals for polymorphisms with statistically significant associations with risk of breast cancer.

Genes and SNPs	Frequency of genotypes^a^		OR (95% CI)^b ^*P^c ^*	FPRP^d ^BFDP^e^
***BMAL1***	No. of cases	No. of controls		
rs2278749				
CC	375	381	1.0	
CT	141	164	0.93 (0.70- 1.22)	
TT	13	30	**0.45 (0.23- 0.90) 0.02**	**0.16 0.21**
			*P*_trend _= 0.07	
***CLOCK ***				
rs3749474				
CC	223	228	1.0	
CT	251	260	1.01 (0.78- 1.31)	
TT	68	108	**0.64 (0.45- 0.92) 0.02**	**0.14 0.11**
			*P*_trend _**= 0.03**	
***AANAT***				
rs3760138				
TT	149	181	1.0	
TG	269	315	1.00 (0.75- 1.32)	
GG	128	106	**1.42 (1.01- 2.01) 0.05**	**0.13 0.18**
			*P*_trend _= 0.07	
rs4238989				
GG	161	203	1.0	
GC	263	288	1.13 (0.86- 1.49)	
CC	120	104	**1.43 (1.01- 2.01) 0.04 **	**0.11 0.16**
			*P*_trend _= 0.13	

^a^Number of cases and controls with successful genotyping data. ^b^Odds ratios (ORs) derived from unconditional logistic regression and adjusted for age at diagnosis (cases) or day of reference (controls), selection period, alcohol use, hormone replacement therapy for the past 2 years, number of children and history of breast cancer in first-degree family (mother or sister). ^c^Pearson's χ^2 ^test (two-sided) comparing frequency of the homozygote variant genotypes with homozygote common genotypes (reference). ^d^The false-positive report probability (FPRP) was calculated as described by Wacholder *et al*. [42]. FPRP < 0.2 was considered noteworthy. ^e^The Bayesian false-discovery probability (BFDP) was calculated as described by Wakefield *et al*. [41]. BFDP < 0.8 was considered noteworthy. For more details on FPRP and BFDP, see Methods section. *P*_trend _values for the Cochran-Armitage trend test were calculated from 2×C tables using frequency of cases and controls.

Compared to the reference group (Table 4), in the highest exposed group with at least four consecutive night shifts, *CLOCK *rs11133373 (OR 2.75, 95% CI 1.07- 7.09), *ROR-b *rs3903529 (OR 1.87, 95% CI 1.0- 3.61), two SNPs in the *AANAT *gene (rs3760138 (OR 1.82, 95% CI 1.13- 2.93) and rs4238989 (OR 1.62, 95% CI 1.0- 2.68)) and *MTNR1B*-rs10830963 (OR 2.48, 95% CI 1.06- 5.8) were associated with increased risk of breast cancer. In the same group, two SNPs in the *CRY2 *gene, rs11038689 (OR 0.42, 95% CI 0.17- 1.01) and rs1401417 (OR 0.31, 95% CI 0.10- 0.94), were associated with reduced risk. In the group with three night shifts, a reduced risk of breast cancer was associated with the presence of one variant allele (heterozygote) or two variant alleles (homozygote) for SNPs in *CLOCK *rs11133373 (OR 0.50, 95% CI 0.25- 0.99), *CLOCK *rs3749474 (OR 0.56, 95% CI 0.31- 0.99); *ROR-b *rs3903529 (OR 0.57, 95% CI 0.34- 0.95), *ROR-b *rs7022435 (OR 0.16, 95% CI 0.05- 0.55); *ROR-b *rs7022435 (OR 0.17, 95% CI 0.03- 0.84); *ROR-b *rs3750420 (OR 0.40, 95% CI 0.18- 0.88) and *MTNR1A *rs 13113549 (OR 0.50, 95% CI 0.28- 0.90).

**Table 4:** Odds ratios and 95% confidence intervals comparing odds of breast cancer associated with each polymorphism among women with less than two consecutive night shifts compared to women with three consecutive night shifts or women with four or more consecutive night shifts.

Genes and SNPs	Number of consecutive night shifts that nurse has worked in all jobs							
	0 to 2 consecutive night shifts		Three consecutive night shifts			≥4 consecutive night shifts		
	Ca^a^/Co^a^	OR (95% CI)^b^	Ca^a^/Co^a^	OR (95% CI)^b ^*P*_inter_^c^	FPRP^d ^BFDP^e^	Ca^a^/Co^a^	OR (95% CI)^b ^*P_inter_*^c^	FPRP^d ^BFDP^e^
***CLOCK ***								
rs11133373								
CC	72/77	1.0 (reference)	71/68	0.82 (0.52 - 1.30)		103/99	1.29 (0.79 - 2.11)	
CG	76/72	0.98 (0.61 -1.57)	58/82	0.77 (0.53 - 1.13)		93/114	0.87 (0.63 - 1.20)	
GG	14/25	1.05 (0.49 - 2.29)	13/29	**0.50 (0.25 **- **0.99) 0.05**	**0.22 0.23**	28/23	1.17 (0.65 - 2.10)	
				*P*_trend_^f ^**= 0.04**			*P*_trend_^f ^= 0.95	
CC	72/77	1.0 (reference)	71/68	0.82 (0.52 - 1.30)		103/99	1.29 (0.79 - 2.11)	
CG	76/72	1.0 (reference)	58/82	0.88 (0.56 - 1.39)		93/114	0.79 (0.48 - 1.30)	
GG	14/25	1.0 (reference)	13/29	0.83 (0.34 - 2.04)		28/23	**2.75 (1.07 **- **7.09) 0.04**	0.30 0.25
								
rs3749474								
CC	67/71	1.0 (reference)	60/66	0.95 (0.57 - 1.57)		96/91	1.14 (0.72 - 1.80)	
CT	77/76	1.12 (0.69-1.81)	73/78	1.01 (0.70 - 1.46)		101/106	1.03 (0.74 - 1.42)	
TT	16/30	0.55 (0.27-1.14)	20/37	**0.56 (0.31 **- **0.99) 0.05**	**0.18 0.21**	32/41	0.85 (0.52 - 1.39)	
				*P*_trend_^f ^= 0.30			*P*_trend_^f ^= 0.54	
CC	67/71	1.0 (reference)	60/66	0.95 (0.57 - 1.57)		96/91	1.14 (0.72 - 1.80)	
CT	77/76	1.0 (reference)	73/78	0.93 (0.58 - 1.48)		101/106	0.98 (0.63 - 1.51)	
TT	16/30	1.0 (reference)	20/37	1.10 (0.46 - 2.61)		32/41	1.35 (0.61 - 3.00)	
								
***CRY2***								
rs11038689								
AA	87/97	1.0 (reference)	84/97	0.99 (0.67 - 1.47)		127/127	1.36 (0.87 - 2.13)	
AG	52/55	1.10 (0.66 - 1.81)	45/57	0.82 (0.54 - 1.26)		68/70	0.94 (0.65 - 1.37)	
GG	8/11	0.86 (0.32 - 2.28)	13/12	1.11 (0.49 - 2.51)		7/20	**0.42 (0.17 **- **1.01) 0.05**	0.31 0.25
				*P*_trend_^f ^= 0.91			*P*_trend_^f ^= 0.39	
AA	87/97	1.0 (reference)	84/97	0.99 (0.67 - 1.47)		127/127	1.36 (0.87 - 2.13)	
AG	52/55	1.0 (reference)	45/57	0.98 (0.58 - 1.68)		68/70	0.82 (0.46 - 1.47)	
GG	8/11	1.0 (reference)	13/12	0.44 (0.09 - 2.06)		7/20	1.44 (0.33 - 6.20)	
								
rs1401417								
CC	78/94	1.0 (reference)	75/95	1.01 (0.65 - 1.58)		119/120	1.27 (0.84 - 1.91)	
CG	46/54	1.10 (0.65-1.86)	40/56	0.76 (0.49 - 1.18)		72/68	1.13 (0.77 - 1.64)	
GG	8/8	1.36 (0.47-3.94)	10/13	0.86 (0.37 - 2.01)		4/17	**0.31 (0.10 **- **0.94) 0.04**	0.37 0.26
				*P*_trend_^f ^= 0.61			*P*_trend_^f ^= 0.78	
CC	78/94	1.0 (reference)	75/95	1.01 (0.65 - 1.58)		119/120	1.27 (0.84 - 1.91)	
CG	46/54	1.0 (reference)	40/56	0.76 (0.42 - 1.37)		72/68	1.11 (0.65 - 1.90)	
GG	8/8	1.0 (reference)	10/13	0.22 (0.18 to 3.74)		4/17	0.25 (0.05 to 1.37)	
								
***NPAS2***								
rs2305160								
GG	75/72	1.0 (reference)	58/71	0.78 (0.48 - 1.28)		105/90	1.16 (0.74 - 1.81)	
GA	61/74	0.82 (0.50 - 1.33)	69/83	0.87 (0.60 - 1.25)		94/104	0.93 (0.67 - 1.29)	
AA	25/27	0.89 (0.46 - 1.72)	21/28	0.73 (0.40 - 1.34)		27/35	0.87 (0.51 - 1.50)	
				*P*_trend_^f ^= 0.26			*P*_trend_^f ^= 0.21	
GG	75/72	1.0 (reference)	58/71	0.78 (0.48 - 1.28)		105/90	1.16 (0.74 - 1.81)	
GA	61/74	1.0 (reference)	69/83	1.06 (0.65 - 1.73)		94/104	1.12 (0.71 - 1.76)	
AA	25/27	1.0 (reference)	21/28	0.76 (0.33 - 1.72)		27/35	0.86 (0.39 - 1.92)	
								
***ROR-b***								
rs3903529								
TT	76/100	1.0 (reference)	81/89	1.29 (0.82 - 2.01)		120/111	1.41 (0.93 - 2.14)	
TA	72/58	1.67 (1.03 - 2.70)	51/73	0.74 (0.49 - 1.10)		81/109	0.81 (0.58 - 1.13)	
AA	10/16	0.89 (0.37 - 2.15)	12/19	0.61 (0.29 - 1.31)		27/15	**1.87 (1.00 **- **3.61) 0.06**	**0.24 0.24**
				*P*_trend_^f ^= 0.60			*P*_trend_^f ^= 0.28	
TT	76/100	1.0 (reference)	81/89	1.29 (0.82 - 2.01)		120/111	1.41 (0.93 - 2.14)	
TA	72/58	1.0 (reference)	51/73	**0.57 (0.34 **- **0.95) 0.03**	**0.12 0.18**	81/109	0.66 (0.41 - 1.05)	
AA	10/16	1.0 (reference)	12/19	0.91 (0.30 - 2.80)		27/15	2.47 (0.83 - 7.34) ***P*_trend _= 0.03**	
								
rs7022435								
GG	89/100	1.0 (reference)	92/101	1.06 (0.69 - 1.62)		124/134	1.06 (0.71 - 1.57)	
GA	65/62	1.21 (0.75 -1.93)	53/63	0.93 (0.61 - 1.40)		82/84	1.05 (0.75 - 1.49)	
AA	8/9	1.12 (0.41 - 3.06)	3/18	**0.16 (0.05 **- **0.55) 0.004**	**0.24 0.23**	18/12	1.55 (0.73 - 3.30)	
				*P*_trend_^f ^= 0.21			*P*_trend_^f ^= 0.31	
rs7022435								
GG	89/100	1.0 (reference)	92/101	1.06 (0.69 - 1.62)		124/134	1.06 (0.71 - 1.57)	
GA	65/62	1.0 (reference)	53/63	0.81 (0.48 - 1.37)		82/84	0.93 (0.58 - 1.49)	
AA	8/9	1.0 (reference)	3/18	**0.17 (0.03 **- **0.84) 0.03**	0.49 0.28	18/12	1.68 (0.44 - 6.33)	
								
rs3750420								
CC	68/87	1.0 (reference)	68/80	1.12 (0.70 - 1.81)		93/112	1.03 (0.66 - 1.61)	
CT	76/72	1.35 (0.84 - 2.18)	68/72	1.11 (0.76 - 1.62)		100/92	1.32 (0.95 - 1.83)	
TT	13/15	1.25 (0.54 - 2.87)	9/25	**0.40 (0.18 **- **0.88) 0.02**	**0.19 0.22**	29/21	1.57 (0.87 - 2.83)	
				*P*_trend_^f ^= 0.58			*P*_trend_^f ^**= 0.04**	
CC	68/87	1.0 (reference)	68/80	1.12 (0.70 - 1.81)		93/112	1-03 (0.66 - 1.61)	
CT	76/72	1.0 (reference)	68/72	0.91 (0.56 - 1.48)		100/92	1.13 (0.72 - 1.76)	
TT	13/15	1.0 (reference)	9/25	0.34 (0.11 - 1.05)		29/21	1.35 (0.51 - 3.58)	
								
***AANAT***								
rs3760138								
TT	38/52	1.0 (reference)	42/57	0.93 (0.51 - 1.70)		69/72	1.19 (0.68 - 2.08)	
TG	86/85	1.17 (0.68 - 2.00)	75/95	0.87 (0.61 - 1.24)		108/135	0.91 (0.67 - 1.24)	
GG	40/42	1.18 (0.63 - 2.21)	33/32	1.23 (0.72 - 2.08)		55/32	**1.82 (1.13 **- **2.93) 0.01**	**0.06 0.14**
				*P*_trend_^f ^**= 0.03**			*P*_trend_^f ^**= 0.04**	
TT	38/52	1.0 (reference)	42/57	0.93 (0.51 - 1.70)		69/72	1.19 (0.68 - 2.08)	
TG	86/85	1.0 (reference)	75/95	0.80 (0.51 - 1.25)		108/135	0.82 (0.54 - 1.23)	
GG	40/42	1.0 (reference)	33/32	1.28 (0.64 - 2.57)		55/32	1.84 (0.94 - 3.62)	
								
rs4238989								
GG	43/60	1.0 (reference)	50/59	1.13 (0.64 - 2.01)		68/84	1.07 (0.62 - 1.83)	
GC	77/82	1.20 (0.72 - 2.02)	66/86	0.87 (0.60 - 1.27)		120/120	1.18 (0.87 - 1.61)	
CC	40/35	1.47 (0.79 - 2.74)	37/39	1.15 (0.70 - 1.88)		43/30	**1.62 (1.00 **- **2.68) 0.06**	**0.19 0.22**
				*P*_trend_^f ^= 0.49			*P*_trend_^f ^**= 0.02**	
GG	43/60	1.0 (reference)	50/59	1.13 (0.64 - 2.01)		68/84	1.07 (0.62 - 1.83)	
GC	77/82	1.0 (reference)	66/86	0.85 (0.53 - 1.37)		120/120	1.11 (0.73 - 1.68)	
CC	40/35	1.0 (reference)	37/39	1.02 (0.52 - 2.01)		43/30	1.40 (0.69 - 2.83)	
								
***MTNR1A***								
rs13113549								
TT	51/60	1.0 (reference)	64/58	1.36 (0.79 - 2.34)		81/78	1.37 (0.82 - 2.27)	
TC	84/88	1.19 (0.72-1.96)	67/80	0.89 (0.61 - 1.30)		102/96	1.08 (0.78 - 1.50)	
CC	26/29	1.12 (0.57-2.20)	20/42	**0.50 (0.28 **- **0.90) 0.02**	**0.11 0.18**	47/56	0.82 (0.54 - 1.26)	
				*P*_trend_^f ^= 0.11			*P*_trend_^f ^= 0.95	
TT	51/60	1.0 (reference)	64/58	1.36 (0.79 - 2.34)		81/78	1.37 (0.82 - 2.27)	
TC	84/88	1.0 (reference)	67/80	0.88 (0.55 - 1.40)		102/96	1.11 (0.73 - 1.71)	
CC	26/29	1.0 (reference)	20/42	0.55 (0.25 - 1.23)		47/56	0.79 (0.40 - 1.59)	
								
***MTNR1B***								
rs10830963								
CC	95/97	1.0 (reference)	76/103	0.74 (0.48 - 1.13)		119/129	0.90 (0.61 - 1.34)	
CG	56/69	0.74 (0.46 - 1.19)	55/60	1.04 (0.69 - 1.56)		86/90	1.06 (0.76 - 1.49)	
GG	7/8	0.76 (0.25 - 2.32)	15/13	1.29 (0.59 - 2.81)		19/8	**2.48 (1.06 **- **5.80) 0.04**	0.26 0.24
				*P*_trend_^f ^= 0.99			*P*_trend_^f ^= 0.35	
CC	95/97	1.0 (reference)	76/103	0.74 (0.48 - 1.13)		119/129	0.90 (0.61 - 1.34)	
CG	56/69	1.0 (reference)	55/60	1.18 (0.70 - 2.00)		86/90	1.23 (0.76 - 1.97)	
GG	7/8	1.0 (reference)	15/13	1.55 (0.38 - 6.37)		19/8	3.51 (0.73 - 16.96)	
								

^a^Number of cases and controls that were successfully genotyped. ^b^OR derived from unconditional logistic regression, including an interaction term between the night shift variable and the SNP genotypes and adjusted for age at diagnosis (cases) or interview (controls), selection period, alcohol use, hormone replacement therapy for the past 2 years, number of children and history of breast cancer in first-degree family members (mother or sister). ^c^*P *interaction was obtained from the interaction term between the SNP genotypes and the night shift variable using frequency of the common genotype in the 0 to 2 night shift group as reference. Only significant or near-significant values are shown. ^d^The false-positive report probability (FPRP) was calculated as described by Wacholder *et al*. [42]. FPRP < 0.2 was considered noteworthy. ^e^The Bayesian false discovery probability (BFDP) was calculated as described by Wakefield *et al*. [41]. BFDP < 0.8 was considered noteworthy. For more details on FPRP and BFDP, see the Methods section. ^f^*P*_trend _values for Cochran-Armitage trend test were calculated from 2×C tables using frequency of genotypes in cases and controls from 0 to 2 night shift group as reference.

The cases and controls were then grouped according to both intensity and duration of night work, that is, maximum number of consecutive nights they had ever worked for at least 5 yr. In subjects with at least four consecutive night shifts, increased risk of breast cancer was associated with variant alleles of *BMAL1 *(rs2290035 (OR 1.91, 95% CI 1.08- 3.37; and OR 1.95, 95% CI 0.97- 3.90) and rs969485 (OR 1.64, 95% CI 1.03- 2.61; and OR 1.63, 95% CI 0.96- 2.76)) and *ROR-b *(rs3750420 (OR 1.60, 95% CI 1.0- 2.56), rs3903529 (OR 2.77, 95% CI 0.98- 7.86)) and *MTNR1B *rs10830963 (OR 9.12, 95% CI 1.14- 73.09). In the group with three consecutive night shifts, the SNP rs11695472 in the *PER2 *gene (OR 2.69, 95% CI 1.08- 6.73) was associated with increased risk of breast cancer. The SNPs *BMAL1 *rs2278749 (OR 0.51, 95% CI 0.29- 0.87), *BMAL2 *rs2306074 (OR 0.30, 95% CI 0.17- 0.75), *CLOCK *rs3749474 (OR 0.24, 95% CI 0.05 to 1.18), *CSNK1E *(rs1534891 (OR 0.55, 95% CI 0.30- 1.01), rs5757037 (OR 0.67, 95% CI 0.45- 0.99)), *NPAS2 *rs17024926 (OR 0.33, 95% CI 0.13- 0.84; and OR 0.24, 95% CI 0.09- 0.67); *PER3 *rs1012477 (OR 0.42, 0.24- 0.72; and OR 0.38, 95% CI 0.18- 0.66), *ROR-b *(rs7022435 (OR 0.25, 95% CI 0.07- 0.89), rs3750420 (OR 0.37, 95% CI 0.14- 0.96; and OR 0.33, 95% CI 0.11- 1.0)) and *MTNR1A *rs131113549 (OR 0.47, 95% CI 0.25- 0.91) were associated with decreased breast cancer risk (Table 5).

**Table 5:** Odds ratios and 95% confidence intervals comparing odds of breast cancer associated with each polymorphism among women with less than two consecutive night shifts for 5 or more years compared to women with three consecutive night shifts or women with four or more consecutive night shifts for 5 or more years

Genes and SNPs	Number of consecutive night shifts nurse has ever worked for 5 or more years							
	0 to 2 consecutive nights		3 consecutive nights			4 or more consecutive nights		
	Ca^a^/Co^a^	OR (95% CI)^b^	Ca^a^/Co^a^	OR (95% CI)^b ^*P*_inter_^c^	FPRP^d ^BFDP^e^	Ca^a^/Co^a^	OR (95% CI)^b ^*P*_inter_^c^	FPRP^d ^BFDP^e^
***ARNTL1/*** ***BMAL1***								
rs2290035								
TT	106/121	1.0 (reference)	40/46	0.98 (0.58 - 1.65)		26/26	0.99 (0.53 - 1.88)	
TA	149/161	1.03 (0.72 - 1.47)	66/71	1.04 (0.71 - 1.51)		46/50	1.02 (0.66 - 1.57)	
AA	50/61	0.95 (0.59 - 1.52)	16/27	0.69 (0.36 - 1.34)		36/21	**1.91 (1.08 **- **3.37) 0.03**	**0.12 0.19**
				*P*_trend_^f ^= 0.60			*P*_trend_^f ^= 0.08	
TT	106/121	1.0 (reference)	40/46	0.98 (0.58 - 1.65)		26/26	0.99 (0.53 - 1.88)	
TA	149/161	1.0 (reference)	66/71	0.99 (0.65 - 1.50)		46/50	1.10 (0.64 - 1.63)	
AA	50/61	1.0 (reference)	16/27	0.75 (0.35 - 1.60)		36/21	**1.95 (0.97 **- **3.90) 0.06**	**0.25 0.24**
								
rs2278749								
CC	212/231	1.0 (reference)	97/92	1.19 (0.84 - 1.70)		66/58	1.26 (0.83 - 1.91)	
CT	86/87	1.19 (0.83 - 1.73)	22/45	**0.51 (0.29 **- **0.87) 0.01**	**0.07 0.15**	33/32	1.10 (0.64 - 1.84)	
TT	7/19	0.40 (0.16 - 0.98)	1/5	0.26 (0.03 - 2.38)		5/6	0.84 (0.25 - 2.82)	
				*P*_trend_^f ^= 0.07			*P*_trend_^f ^= 0.52	
CC	212/231	1.0 (reference)	97/92	1.19 (0.84 - 1.70)		66/58	1.26 (0.83 - 1.91)	
CT	86/87	1.0 (reference)	22/45	**0.47 (0.25 **- **0.88) 0.02**	**0.12 0.18**	33/32	0.99 (0.55 - 1.79)	
TT	7/19	1.0 (reference)	1/5	1.33 (0.10 - 17.78)		5/6	2.68 (0.48 - 15.01)	
								
rs969485								
AA	161/189	1.0 (reference)	51/74	0.83 (0.54 - 1.28)		41/50	0.92 (0.57 - 1.49)	
AG	105/117	1.05 (0.74 - 1.49)	58/55	1.23 (0.82 - 1.85)		50/35	**1.64 (1.03 **- **2.61) 0.04**	**0.12 0.18**
GG	21/20	1.07 (0.54 - 2.09)	8/8	1.40 (0.50 - 3.94)		9/7	1.50 (0.53 - 4.22)	
				*P*_trend_^f ^= 0.49			*P*_trend_^f ^**= 0.05**	
AA	161/189	1.0 (reference)	51/74	0.83 (0.54 - 1.28)		41/50	0.92 (0.57 to 1.49)	
AG	105/117	1.0 (reference)	58/55	1.50 (0.72 - 1.85)		50/35	**1.63 (0.96 **- **2.76) 0.07**	**0.21 0.23**
GG	21/20	1.0 (reference)	8/8	1.35 (0. 37 - 4.88)		9/7	1.60 (0.38 - 6.74)	
								
***ARNTL2/*** ***BMAL2 ***								
rs2306074								
TT	143/144	1.0 (reference)	58/63	0.91 (0.59 - 1.43)		51/53	0.97 (0.61 - 1.53)	
TC	134/163	0.84 (0.60 - 1.17)	60/68	0.97 (0.66 - 1.42)		50/40	1.36 (0.86 - 2.13)	
CC	37/39	1.04 (0.61 - 1.76)	6/21	**0.30 (0.17 **- **0.75) 0.01**	**0.18 0.21**	9/9	1.03 (0.39 - 2.72)	
				*P*_trend_^f ^**= **0.06			*P*_trend_^f ^= 0.51	
TT	143/144	1.0 (reference)	58/63	0.91 (0.59 - 1.43)		51/53	0.97 (0.61 - 1.53)	
TC	134/163	1.0 (reference)	60/68	1.06 (0.68 - 1.64)		50/40	1.37 (0.83 - 2.26)	
CC	37/39	1.0 (reference)	6/21	**0.24 (0.08 **- **0.77) 0.02**	0.31 0.25	9/9	0.99 (0.32 - 3.11)	
								
***CLOCK ***								
rs3749474								
CC	123/131	1.0 (reference)	52/55	0.97 (0.68 - 1.37)		48/42	1.08 (0.73 - 1.59)	
CT	148/152	1.04 (0.74 - 1.47)	56/63	0.87 (0.46 - 1.63)		47/45	1.84 (0.92 - 3.69)	
TT	39/66	0.60 (0.37 - 0.97)	16/29	**0.24 (0.05 **- **1.18) 0.07**	0.56 0.29	13/13	3.15 (0.62 - 16.14)	
				*P*_trend_^f ^= 0.24			*P*_trend_^f ^= 0.63	
CC	123/131	1.0 (reference)	52/55	0.97 (0.68 - 1.37)		48/42	1.08 (0.73 - 1.59)	
CT	148/152	1.0 (reference)	56/63	0.64 (0.30 - 1.36)		47/45	1.44 (0.63 - 3.31)	
TT	39/66	1.0 (reference)	16/29	0.30 (0.05 - 1.77)		13/13	2.12 (0.30 - 15.15)	
								
***CSNK1E ***								
rs1534891								
GG	241/258	1.0 (reference)	98/112	0.93 (0.66 - 1.309)		82/76	1.12 80.77 - 1.62)	
GA	62/77	0.83 (0.57 - 1.23)	17/35	**0.55 (0.30 **- **1.01) 0.05**	**0.21 0.23**	25/25	1.09 (0.61 - 1.95)	
AA	5/9	0.51 (0.16 - 1.60)	2/1	2.00 (0.17 - 23.92)		1/2	0.39 (0.03 - 4.68)	
				*P*_trend_^f ^= 0.13			*P*_trend_^f ^= 0.66	
GG	241/258	1.0 (reference)	98/112	0.93 (0.66 - 1.309)		82/76	1.12 80.77 - 1.62)	
GA	62/77	1.0 (reference)	17/35	0.63 (0.31 - 1.28)		25/25	1.11 (0.56 - 2.21)	
AA	5/9	1.0 (reference)	2/1	2.58 (0.14 - 91.16)		1/2	2.84 (0.08 - 96.47)	
								
rs5757037								
CC	126/154	1.0 (reference)	49/57	1.10 (0.68 - 1.76)		41/38	1.28 (0.75 - 2.70)	
CT	137/140	1.11 (0.79 - 1.58)	51/77	**0.67 (0.45 **- **0.99) 0.05**	**0.13 0.18**	49/45	1.14 (0.74 - 1.77)	
TT	43/43	1.04 (0.63 - 1.71)	14/16	0.90 (0.43 - 1.90)		16/16	1.00 (0.49 - 2.05)	
				*P*_trend_^f ^= 0.57			*P*_trend_^f ^= 0.22	
CC	126/154	1.0 (reference)	49/57	1.10 (0.68 - 1.76)		41/38	1.28 (0.75 - 2.70)	
CT	137/140	1.0 (reference)	51/77	0.65 (0.42 - 1.02)		49/45	1.08 (0.66 - 1.75)	
TT	43/43	1.04 (0.63- 1.71)	14/16	0.89 (0.38 - 2.10)		16/16	0.97 (0.42 - 2.23)	
								
***NPAS2***								
rs2305160								
GG	136/134	1.0 (reference)	52/57	0.86 (0.54 - 1.36)		50/42	1.15 (0.70 - 1.88)	
GA	130/151	0.84 (0.59 - 1.18)	51/69	0.84 (0.56 - 1.25)		43/41	1.10 (0.70 - 1.74)	
AA	41/56	0.72 (0.45 - 1.18)	18/21	0.87 (0.45 - 1.69)		14/13	1.22 (0.56 - 2.64)	
				*P*_trend_^f ^= 0.21			*P*_trend_^f ^= 0.79	
rs2305160								
GG	136/134	1.0 (reference)	52/57	0.86 (0.54 - 1.36)		50/42	1.15 (0.70 - 1.88)	
GA	130/151	1.0 (reference)	51/69	0.95 (0.61 - 1.50)		43/41	1.19 (0.72 - 1.96)	
AA	41/56	1.0 (reference)	18/21	1.10 (0.50 - 2.40)		14/13	1.43 (0.58 - 3.52)	
								
rs17024926								
TT	110/133	1.0 (reference)	60/66	1.22 (0.78 - 1.92)		36/45	1.03 (0.61 - 1.75)	
TC	148/178	1.08 (0.76 - 1.52)	55/64	0.98 (0.66 - 1.47)		54/46	1.23 (0.80 - 1.89)	
CC	47/38	1.51 (0.90 - 2.52)	6/21	**0.33 (0.13 **- **0.84) 0.020**	**0.24 0.23**	17/10	1.89 (0.85 - 4.23)	
				*P*_trend_^f ^= 0.28			*P*_trend_^f ^**= 0.05**	
TT	110/133	1.0 (reference)	60/66	1.22 (0.78 - 1.92)		36/45	1.03 (0.61 - 1.75)	
TC	148/178	1.0 (reference)	55/64	1.01 (0.65 - 1.57)		54/46	1.28 (0.81 - 2.04)	
CC	47/38	1.0 (reference)	6/21	**0.24 (0.09 **- **0.67) 0.006**	**0.19 0.22**	17/10	1.49 (0.56 - 3.95)	
								
***PER2***								
rs11695472								
AA	173/187	1.0 (reference)	68/87	0.87 (0.59 - 1.29)		63/48	1.37 (0.88 - 2.14)	
AC	117/143	0.90 (0.65 - 1.25)	38/54	0.74 (0.47 - 1.16)		36/44	0.87 (0.54 - 1.39)	
CC	22/21	1.24 (0.64 - 2.39)	16/7	**2.69 (1.08 **- **6.73) 0.03**	0.28 0.25	9/8	1.32 (0.48 - 3.65)	
				*P*_trend_^f ^= 0.93			*P*_trend_^f ^= 0.79	
AA	173/187	1.0 (reference)	68/87	0.87 (0.59 - 1.29)		63/48	1.37 (0.88 - 2.14)	
AC	117/143	1.0 (reference)	38/54	0.83 (0.50 - 1.39)		36/44	0.93 (0.55 - 1.55)	
CC	22/21	1.0 (reference)	16/7	1.91 (0.59 - 6.22)		9/8	0.83 (0.22 - 3.14)	
								
***PER3 ***								
rs1012477								
GG	203/237	1.0 (reference)	88/97	1.12 (0.77 - 1.58)		66/73	1.02 (0.69 - 1.51)	
GC	95/100	1.10 (0.78 - 1.56)	22/51	**0.42 (0.24 **- **0.72) 0.002**	**0.02 0.08**	35/24	1.56 (0.91 - 2.68)	
CC	9/7	1.69 (0.58 - 4.93)	3/1	3.42 (0.35 - 33.40)		3/2	1.90 (0.31 - 11.58)	
				*P*_trend_^f ^= 0.15			*P*_trend_^f ^= 0.08	
GG	203/237	1.0 (reference)	88/97	1.12 (0.77 - 1.58)		66/73	1.02 (0.69 - 1.51)	
GC	95/100	1.0 (reference)	22/51	**0.38 (0.18 **- **0.66) 0.001**	**0.01 0.06**	35/24	1.46 (0.79 - 2.72)	
CC	9/7	1.0 (reference)	3/1	3.00 (0.12 - 77.00)		3/2	1.30 (0.06 - 28.93)	
								
***ROR-b***								
rs3903529								
TT	162/183	1.0 (reference)	64/70	1.09 (0.71 - 1.66)		51/47	1.20 (0.75 - 1.93)	
TA	123/131	1.08 (0.77 - 1.51)	41/62	0.75 (0.49 - 1.15)		40/47	0.92 (0.59 - 1.46)	
AA	22/27	0.98 (0.53 - 1.82)	13/18	0.76 (0.36 - 1.59)		14/5	**2.77 (0.98 **- **7.86) 0.056**	0.38 0.27
				*P*_trend_^f ^= 0.26			*P*_trend_^f ^= 0.21	
TT	162/183	1.0 (reference)	64/70	1.09 (0.71 - 1.66)		51/47	1.20 (0.75 - 1.93)	
TA	123/131	1.0 (reference)	41/62	0.72 (0.44 - 1.16)		40/47	0.94 (0.57 - 1.55)	
AA	22/27	1.0 (reference)	13/18	0.78 (0.30 - 2.05)		14/5	3.07 (0.84 - 11.18)	
								
rs7022435								
GG	178/194	1.0 (reference)	72/85	0.97 (0.65 - 1.43)		55/56	1.05 (0.67 - 1.64)	
GA	115/128	1.00 (0.72 - 1.40)	44/49	1.02 (0.65 - 1.60)		41/32	1.38 (0.84 - 2.26)	
AA	15/19	0.93 (0.45 - 1.90)	3/12	**0.25 (0.07 **- **0.89) 0.03**	0.41 0.27	11/8	1.34 (0.52 - 3.41)	
				*P*_trend_^f ^= 0.25			*P*_trend_^f ^= 0.15	
GG	178/194	1.0 (reference)	72/85	0.97 (0.65 - 1.43)		55/56	1.05 (0.67 - 1.64)	
GA	115/128	1.0 (reference)	44/49	1.03 (0.62 - 1.70)		41/32	1.34 (0.78 - 2.92)	
AA	15/19	1.0 (reference)	3/12	0.29 (0.06 - 1.33)		11/8	2.18 (0.55 - 8.61)	
								
rs3750420								
CC	136/166	1.0 (reference)	52/66	0.99 (0.63 - 1.55)		41/47	1.02 (0.61 - 1.68)	
CT	136/143	1.20 (0.86 - 1.68)	60/58	1.24 (0.83 - 1.86)		48/35	**1.60 (1.00 **- **2.56) 0.05**	**0.15 0.20**
TT	28/31	1.18 (0.67 - 2.08)	6/18	**0.37 (0.14 **- **0.96) 0.04**	0.31 0.25	17/12	1.52 (0.71 - 3.28)	
				*P*_trend_^f ^= 0.82			*P*_trend_^f ^**= 0.03**	
CC	136/166	1.0 (reference)	52/66	0.99 (0.63 - 1.55)		41/47	1.02 (0.61 - 1.68)	
CT	136/143	1.0 (reference)	60/58	1.13 (0.72 - 1.77)		48/35	1.47 (0.88 - 2.45)	
TT	28/31	1.0 (reference)	6/18	**0.33 (0.11 **- **1.00) 0.05**	0.39 0.27	17/12	1.39 (0.54 - 3.60)	
***MTNR1A ***								
rs13113549								
TT	113/109	1.0 (reference)	48/54	0.94 (0.57 - 1.53)		35/33	1.09 (0.62 - 1.93)	
TC	142/155	0.94 (0.66 - 1.35)	59/60	1.08 (0.72 - 1.61)		52/49	1.08 (0.71 - 1.65)	
CC	55/75	0.75 (0.48 - 1.18)	16/34	**0.47 (0.25 **- **0.91) 0.02**	**0.15 0.20**	22/18	1.24 (0.65 - 2.39)	
				*P*_trend_^f ^= 0.08			*P*_trend_^f ^= 0.71	
TT	113/109	1.0 (reference)	48/54	0.94 (0.57 - 1.53)		35/33	1.09 (0.62 - 1.93)	
TC	142/155	1.0 (reference)	59/60	1.03 (0.66 - 1.61)		52/49	1.09 (0.68 - 1.75)	
CC	55/75	1.0 (reference)	16/34	0.57 (0.27 - 0.19)		22/18	1.28 (0.61 - 2.68)	
								
***MTNR1B***								
rs10830963								
CC	176/195	1.0 (reference)	63/84	0.83 (0.58 - 1.23)		51/50	1.01 (0.64 - 1.61)	
CG	105/123	0.86 (0.61 - 1.21)	46/51	1.06 (0.69 - 1.64)		46/45	1.13 (0.73 - 1.75)	
GG	21/21	1.05 (0.55 - 2.02)	11/7	1.59 (0.59 - 4.25)		9/1	**9.12 (1.14 **- **73.09) 0.04**	0.59 0.29
				*P*_trend_^f ^= 0.76			*P*_trend_^f ^**= **0.10	
CC	176/195	1.0 (reference)	63/84	0.83 (0.58 - 1.23)		51/50	1.01 (0.64 - 1.61)	
CG	105/123	1.0 (reference)	46/51	1.17 (0.71 - 1.93)		46/45	1.26 (0.76 - 2.08)	
GG	21/21	1.0 (reference)	11/7	1.44 (0.43 - 4.86)		9/1	9.45 (0.97 - 91.82)	

^a^Number of cases and controls that were successfully genotyped. ^b^OR derived from unconditional logistic regression, including an interaction term between the night shift variable and the SNP genotypes, and adjusted for age at diagnosis (cases) or interview (controls), selection period, alcohol use, hormone replacement therapy for the past 2 years, number of children and history of breast cancer in first-degree family members (mother or sister). ^c^*P *interaction was obtained from the interaction term between the SNP genotypes and the night shift variable using frequency of the common genotype in the 0 to 2 night shift group as reference. Only significant or near-significant values are shown. ^d^The false-positive report probability (FPRP) was calculated as described by Wacholder *et al*. [42]. FPRP < 0.2 was considered noteworthy. ^e^The Bayesian false discovery probability (BFDP) was calculated as described by Wakefield *et al*. [41]. BFDP < 0.8 was considered noteworthy. For more details on FPRP and BFDP, see the Methods section. ^f^*P*_trend _values for Cochran-Armitage trend test were calculated from 2×C tables using frequency of genotypes in cases and controls from 0 to 2 night shift group as reference.

After applying FPRP and BFDP tests, in subjects with four or more night shifts (Table 4), increased risk of breast cancer associated with homozygote carriers of the variant alleles of SNPs rs3760138 and rs4238989 in the *AANAT *gene was found to be noteworthy. In subjects with three consecutive night shifts, the odds of reduced risk of breast cancer associated with carriage of variant alleles of SNPs in *CLOCK *(rs3749474), *ROR-b *(rs3903529, rs3750420) and *MTNR1A *(rs131113549) were found noteworthy. With respect to both intensity and duration of night shift work for at least 5 yr (Table 5), in subjects with at least four consecutive night shifts, increased risk of breast cancer was associated with variant alleles of *BMAL1 *(rs2290035, rs969485) and *ROR-b *(rs3750420). In the subjects with three night shifts, SNPs in *BMAL1 *(rs2278749), *BMAL2 *(rs2306074), *CSNK1E *(rs5757037), *NPAS2 *(rs17024926), *PER3 *(rs1012477) and *MTNR1A *(rs131113549) were associated with decreased breast cancer risk. Haplotype analysis of the polymorphisms localized on the same chromosome did not result in any significant associations (data not shown).

## Discussion

In the main effects analysis, two SNPs in *BMAL1 *and *CLOCK *were associated with risk of breast cancer. There are no functional data to explain how each SNP in each of the genes may affect the risk for breast cancer. SNPs may change transcription binding affinity of the BMAL1/CLOCK complex that is necessary for expression of the target genes. For example, a reduced affinity of BMAL1/CLOCK to *CLOCK *promoter due to polymorphisms in these genes may lead to lower expression of CLOCK proteins. A reduction in the levels of another core circadian protein (CRY2) in breast epithelial cells in culture has been shown to lead to higher accumulation of DNA damage after exposure to chemical mutagens [48,49]. Downregulation of CLOCK has also been reported to cause alterations in expression of genes involved in breast carcinogenesis [50].

Several SNPs in the core circadian genes had noteworthy associations with breast cancer risk. Women with the highest number of successive night shifts and carrying at least one variant allele of SNPs in the two core circadian genes *BMAL1 *(rs2290035, rs969485) and *ROR-b *(rs3903529, rs3750420) had a noteworthy increased risk of breast cancer. Although not noteworthy, there was also a 2.75 increased risk of breast cancer in women who had worked four or more night shifts and carrying two variant G alleles of *CLOCK *rs111133373 compared to women with the same genotype in the reference group. Interestingly, women with three successive night shifts and carrying the same SNPs and genotypes had a reduced risk of breast cancer, indicating a protective role for these genetic variants. In addition, women in this exposure group carrying at least one variant allele of several SNPs in the other core circadian genes such as *BMAL2*, *CSNK1E*, *NPAS2 *and *PER3 *had a noteworthy reduced risk of breast cancer. For comparison, only two SNPs in the *CRY2 *gene (rs11038689 and rs1401417) had protective effects in women with four or more night shifts, though this effect was not noteworthy.

Many of the associations, such as *PER3 *(rs1012477), *CSNK1E *(rs1534891) and *CLOCK *(rs11133373, rs3749474), are novel in relation to breast risk but may not be specific to breast cancer, because they have also been found to be associated with prostate and colorectal cancer risk [47,51]. It might be that these SNPs affect expression of the respective genes. Current results from the available literature on the functionality of other SNPs in these genes are not conclusive. For example, it has previously been seen that, in healthy nurses and midwives who worked either day or rotating night shifts, there were no differences between *BMAL1*, *CLOCK*, *CRY1*, *CRY2*, *PER1*, *PER2 *and *PER3 *gene expression levels in peripheral blood leukocytes [52]. In the same subjects, however, polymorphisms of the circadian genes *BMAL1 *(rs2279287), *CLOCK *(rs1801260), *PER1 *(rs2735611), *PER2 *(rs2304672), *PER3 *(rs10462020), *CRY1 *(rs8192440) and *CRY2 *(rs10838527, rs10838527) were not found to be different between shift workers and day-only workers [53].

In a recent study by Monsees *et al*. [54], the SNP rs2305160 (Ala394Thr) in the *NPAS2 *gene was found to be associated with reduced breast cancer risk in women with less than 2 yr of shift work. We were not able to confirm such an association in our study; however, for another SNP, rs17024926 in the *NPAS2 *gene, we found a reduced risk of breast cancer in shift workers who had worked three night shifts for at least 5 yr (Table 5). The reduced risk was associated with the variant homozygote genotype where carriers of the CC variant genotype had reduced risk of breast cancer compared to subjects who had worked no or few night shifts carrying either the variant CC genotypes or the common TT genotype (Table 5). The *NPAS2 *rs17024926 has been found to be associated with risk of prostate cancer [47]. This SNP was not genotyped in the study by Monsees *et al*. [54]; however, the reasons why the results of our study and those of Monsees *et al*. differ for Ala394Thr variant are not known. This difference could not be due to differences in MAF in the two populations, first because the MAF 0.37 for this SNP in our population (Additional File 2, Table S2) is similar to the MAF 0.34 in the study by Monsees *et al*., and second because we regenotyped this SNP in our subjects and again found no associations. There could be several other possibilities, such as differences in study design, population differences in incidence of breast cancer, exposure metrics and the fact that breast cancer is known to be a genetically heterogeneous disease. Nevertheless, both studies support a role of circadian genes in breast cancer in shift workers, which warrants further confirmation and validation in future studies.

Increased risk of breast cancer in women with four or more night shifts was associated with *ROR-b *SNPs rs3903529 and rs3750420 (heterozygotes), whereas women with three night shifts and carrying a homozygote variant of rs7022435 and rs3750420 had reduced risk. The three SNPs are located in intron 1 downstream of the exon 1 in the *ROR-b *transcripts. These SNPs are part of a haplotype block that spans the region containing the promoter region and the exon 1 (55 kb upstream to 6 kb downstream) of the gene. This haplotype has also been associated with an increased risk of human bipolar syndrome [55], but we were not able to find such an association with breast cancer risk (data not shown). If these SNPs, or others in linkage disequilibrium with them, may represent susceptibility variants for breast cancer, it is possible they do so by altering the gene expression of the *ROR-b *gene. The ROR proteins are involved in regulation of circadian rhythms by activating transcription of the core circadian *BMAL1 *gene [56].

Two polymorphisms in the *CRY2 *gene (rs11038689 and rs1401417) had protective effects in women with four or more night shifts. The biological functions of these SNPs are not known. These polymorphisms have been found to be associated with postmenopausal breast cancer risk, with significant effect modification by menopausal status where this association was evident only in women with ER- and/or PR-negative breast tumors but not ER- and/or PR-positive tumors [57]. This may indicate that these polymorphisms may influence breast cancer risk through estrogen and progesterone signaling pathways. ERs and PRs are important for reproductive performance in women. Kovanen *et al*. [46] showed that women with *BMAL1 *rs2278749 TT genotypes had more pregnancies and miscarriages, whereas *NPAS2 *rs11673746 T carriers had fewer miscarriages, indicating a possible role of these polymorphisms affecting age at first birth and also number of children, both of which are established risk factors for breast cancer [58].

In the genes controlling melatonin biosynthesis (*AANAT*) or melatonin receptors (*MTNR1A *and *MTNR1B*), several SNPs were associated with increased risk of breast cancer. In the main effects analysis, the variant genotypes of both of the *AANAT *polymorphisms were associated with increased breast cancer risk. However, the risk associated with the rs3760138 GG genotype was 40% higher in the most exposed group with four or more night shifts (1.82 vs 1.42). These two SNPs are located in the regulatory regions of the *AANAT *gene which could potentially affect the expression of the *AANAT *gene. The *AANAT *gene product is an acetyltransferase that regulates melatonin biosynthesis during night and day periods by pineal gland. Polymorphisms in this gene have also been associated with sleep disorders, but there are no data on the association between these polymorphisms and breast cancer risk. The levels of melatonin may also be affected by variants in the genes that encode melatonin receptors 1A and 1B. The M*TNR1B *rs10830963 was associated with increased risk in the high-level exposed group of women carrying two variant alleles, without being noteworthy by FPRP and BFDP. The variant alleles of *MTNR1A *rs113113549 polymorphisms were associated with decreased risk of breast cancer in the women with three consecutive night shifts. Night shift workers have been shown to have substantially reduced melatonin levels during night work and daytime sleep, and levels remain low even when a night shift worker sleeps at night [22].

### Strengths and limitations of the study

Our results indicate that some SNPs of the core circadian or melatonin signaling pathways, in combination with night work, may affect breast cancer risk. These findings are consistent with the results of recent studies, including our previous study of the same nurses, suggesting a higher risk with longer duration of intense night shift work per week [7,9,10]. The reported associations are biologically plausible, as the polymorphisms in the genes investigated could influence gene expression, protein function and protein-protein interactions, all of which may affect several known risk factors for breast cancer. However, this is a medium-sized, exploratory study highlighting the need for investigation of possible combinatory effects of genetic polymorphisms and night shift work in breast cancer. The results should be interpreted with caution, and larger studies are needed to confirm the associations found here.

The study may have a number of limitations. A larger reference group without any night shift work is needed. Our decision to use women with maximum of two consecutive night shifts as the reference group was made to ensure a sufficient number of subjects for each genotype. Furthermore, the metrics of exposure to night work used in our study are different from those used in other studies; therefore, the results may not be comparable. We have selected a limited number of polymorphisms, and, to cover all SNPs in the core circadian and melatonin signaling pathways, a much larger study (that is, GWAS) is warranted. The selection of genes and polymorphisms differs from other studies as well and may have affected the outcomes. Finally, although we set very low prior probabilities for multiple testing, it is still possible that some associations may not be true positive or negative findings.

## Conclusion

We have found a number of polymorphisms in a number of core circadian or melatonin biosynthesis and/or bioavailability pathway genes that could be associated with breast cancer risk in shift workers. The associations seem biologically plausible because many of the polymorphisms may alter gene expression, mRNA stability or protein function. Future studies investigating the genetic and epigenetic functionality of these SNPs, as well as epigenetic changes in the shift workers, are warranted to understand exact biological mechanisms.

## Abbreviations

AANAT: Arylalkylamine *N*-acetyltransferase; ARNTL: Aryl hydrocarbon receptor nuclear translocator-like; CGEMS: Cancer genetic marker of susceptibility; CIGENE: Centre for Integrative Genetics; CLOCK: Circadian locomotor output cycle kaput; CRY: Cryptochrome; CSNK1E: Casein kinase 1; ER: Estrogen receptor; GWAS: Genome-wide association study; H-W: Hardy-Weinberg; LAN: Light at night; MAF: Minor allele frequency; MTNR: Melatonin receptor; NPAS2: Neuronal PAS domain protein 2; PER: Period; ROR: RAR-related orphan receptor; SNP: Single-nucleotide polymorphism

## Competing interests

The authors declare that they have no competing interests.

## Authors' contributions

SZ, AH and KHA were involved in designing and conducting the genetic studies, genotyping and data analysis and wrote the first and revised drafts of the manuscript. JASL, HK and KK were responsible for the design, coordination and execution of the case-control study and were also involved in revising the manuscript. All authors read and approved the final manuscript.

## Supplementary Material

Additional file 1 **Legend to Supplementary Figure 1: The flow-chart showing study design**. Click here for file

Additional file 2 **Supplementary Tables S1-Table S2**. Supplementary Table S1: Full name of the circadian genes and their protein functions. Supplementary Table S2: The circadian genes, SNPs, base change, MAF/GMAF, Hardy-Weinberg values and genotyping rate Click here for file
