# Supplementary material for: Analysis of polymorphisms in the circadian-related genes and breast cancer risk in Norwegian nurses working night shifts
Source: Breast Cancer Res. 2013 Jul 3;15(4):R53. doi: 10.1186/bcr3445 (PMC3978690; doi:10.1186/bcr3445)
Supplement: Additional file 1 — Legend to Supplementary Figure 1: The flow-chart showing study design. [file bcr3445-S1.DOC]

**Supplementary Figure 1: The flow-chart showing study design**

**The Norwegian Nurses Cohort (nurses graduated 1914-1985, N=49402)**

**Breast cancer cases Cancer free controls**

**Cases diagnosed with invasive breast cancer**

**between 1990-2007 (n=1132)**

**Cases alive by Feb. 2009 (n= 943) Age-matched cancer-free nurses alive by 2009 (n=1384)**

**Cases interviewed (n= 699) Nurses met occupational exposure and interviewed (n=895)**

**Cases consented to send saliva (n=563) Nurses consented to send saliva samples for genetic analysis (619)**
