# Supplementary material for: Analysis of polymorphisms in the circadian-related genes and breast cancer risk in Norwegian nurses working night shifts
Source: Breast Cancer Res. 2013 Jul 3;15(4):R53. doi: 10.1186/bcr3445 (PMC3978690; doi:10.1186/bcr3445)
Supplement: Additional file 2 — Supplementary Tables S1-Table S2. Supplementary Table S1: Full name of the circadian genes and their protein functions. Supplementary Table S2: The circadian genes, SNPs, base change, MAF/GMAF, Hardy-Weinberg values and genotyping rate [file bcr3445-S2.PDF]

Supplementary Table S1: Full name of the circadian genes and their protein functions

Full gene name(s)	Pathway	Chromosome and location	Protein function
Aryl hydrocarbon receptor nuclear translocator-like 1 (*ARNTL1) also known as Brain and muscle ARNTL-like 1 (BMAL1)*	Core circadian	11p15	The protein encoded by this gene is a basic helix-loop-helix protein that forms a heterodimer with CLOCK. This complex binds an E-box upstream of the PER1 gene, activating this gene and possibly other circadian rhythym-associated genes. Three transcript variants encoding two different isoforms have been found for this gene.
Aryl hydrocarbon receptor nuclear translocator-like 2 (*ARNTL2) also known as Brain and muscle ARNTL-like 2 (BMAL2)*	Core circadian	12p12	This gene encodes a basic helix-loop-helix transcription factor belonging to the PAS (PER, ARNT, SIM) superfamily. This protein forms a transcriptionally active heterodimer with the circadian CLOCK protein, the structurally related MOP4, and hypoxia-inducible factors, such as HIF1alpha. Consistent with its role as a biologically relevant partner of circadian and hypoxia factors, this protein is coexpressed in regions of the brain such as the thalamus, hypothalamus, and amygdala.
Circadian locomoter output cycles kaput protein *(CLOCK)*	Core circadian	4q12	This gene encodes a protein that belongs to the basic helix-loop-helix family of transcription factors. A similar protein in mice is a circadian regulator that acts as a transcription factor and forms a heterodimer with aryl hydrocarbon receptor nuclear translocator-like to activate transcription of mouse period 1.
Cryptochrome 1 (photolyase-like) (*CRY1)*	Core circadian	12q23-q24.1	CRY1 is essential for proper circadian timing and is a key component of the circadian regulatory feedback loop.
Cryptochrome 2 (photolyase-like) (*CRY2)*	Core circadian	11p11.2	CRY2 is essential for proper circadian timing and is a key component of the circadian regulatory feedback loop.
Casein kinase 1, epsilon *(CSNK1E)*	Core circadian	22q13.1	The protein encoded by this gene is a serine/threonine protein kinase and a member of the casein kinase I protein family, whose members have been implicated in the control of cytoplasmic and nuclear

			processes, including DNA replication and repair. The encoded protein is found in the cytoplasm as a monomer and can phosphorylate a variety of proteins, including itself. Two transcript variants encoding the same protein have been found for this gene.
Neuronal PAS domain protein 2 *(NPAS2)*	Core circadian	2q11.2	The protein encoded by this gene is also a member of the basic helix-loop-helix -PAS family of transcription factors. It functions as a part of the molecular clock operative in mammalians involved in diurnal oscillations.
PERIOD 1 (*PER1)*	Core circadian	17p13.1	This gene is a member of the Period family of genes and is expressed in a circadian pattern in the suprachiasmatic nucleus, the primary circadian pacemaker in the mammalian brain. Genes in this family encode components of the circadian rhythms of locomotor activity, metabolism, and behavior. Circadian expression in the suprachiasmatic nucleus continues in constant darkness, and a shift in the light/dark cycle evokes a proportional shift of gene expression in the suprachiasmatic nucleus.
PERIOD *2 (PER2)*	Core circadian	2q37.3	This gene is a member of the Period family of genes and is expressed in a circadian pattern in the suprachiasmatic nucleus, the primary circadian pacemaker in the mammalian brain. Genes in this family encode components of the circadian rhythms of locomotor activity, metabolism, and behavior. Circadian expression in the suprachiasmatic nucleus continues in constant darkness, and a shift in the light/dark cycle evokes a proportional shift of gene expression in the suprachiasmatic nucleus.
PERIOD *3 (PER3)*	Core circadian	1p6.3	This gene is a member of the Period family of genes and is expressed in a circadian pattern in the suprachiasmatic nucleus, the primary circadian pacemaker in the mammalian brain. Genes in this family encode components of the circadian rhythms of locomotor activity, metabolism, and behavior. Circadian expression in the suprachiasmatic nucleus continues in constant darkness, and a shift in the light/dark cycle evokes a proportional shift of gene expression in the suprachiasmatic nucleus.

RAR(retinoic acid receptor)-related orphan receptor A *(ROR-a)*	Core circadian	15q22.2	The protein encoded by this gene is a member of the NR1 subfamily of nuclear hormone receptors. It is a DNA-binding protein that can bind as a monomer or as a homodimer to hormone response elements upstream of several genes to enhance the expression of those genes.
RAR(retinoic acid receptor)-related orphan receptor B (*ROR-b)*	Core circadian	9q22	The protein encoded by this gene is a member of the NR1 subfamily of nuclear hormone receptors. It is a DNA-binding protein that can bind as a monomer or as a homodimer to hormone response elements upstream of several genes to enhance the expression of those genes.
TIMELESS	Core circadian	12q13.3	
Aralkylamine N-acetyltransferase (*AANAT)*	Melatonin synthesis	17q25	The protein encoded by this gene belongs to the acetyltransferase superfamily. It is the ultimate enzyme in melatonin synthesis and controls the night/day rhythm in melatonin production in the vertebrate pineal gland. Melatonin is essential for the function of the circadian clock that influences activity and sleep. This enzyme is regulated by cAMP-dependent phosphorylation that promotes its interaction with 14-3-3 proteins and thus protects the enzyme against proteasomal degradation. This gene may contribute to numerous genetic diseases such as delayed sleep phase syndrome. Alternatively spliced transcript variants encoding different isoforms have been found for this gene.
Melatonin receptor 1A (*MTNR1A/MT1)*	Melatonin receptor type1	4q35.1	This gene encodes one of two high affinity forms of a receptor for melatonin, the primary hormone secreted by the pineal gland. This receptor is a G-protein coupled, 7-transmembrane receptor that is responsible for melatonin effects on mammalian circadian rhythm and reproductive alterations affected by day length. The receptor is an integral membrane protein that is readily detectable and localized to two specific regions of the brain. The hypothalamic suprachiasmatic nucleus appears to be involved in circadian rhythm while the hypophysial pars tuberalis may be responsible for the reproductive effects of melatonin.

Melatonin receptor 1B (*MTNR1B/MT2)*	Melatonin receptor 2	11q21-q22	This gene encodes one of two high affinity forms of a receptor for melatonin, the primary hormone secreted by the pineal gland. This receptor is a G-protein coupled, 7-transmembrane receptor that is responsible for melatonin effects on mammalian circadian rhythm and reproductive alterations affected by day length. The receptor is an integral membrane protein that is readily detectable and localized to two specific regions of the brain. The hypothalamic suprachiasmatic nucleus appears to be involved in circadian rhythm while the hypophysial pars tuberalis may be responsible for the reproductive effects of melatonin.
OPN4/MOP	Melatonin synthesis	10q22	Opsins are members of the guanine nucleotide-binding protein (G protein)-coupled receptor superfamily. This gene encodes a photoreceptive opsin protein that is expressed within the ganglion and amacrine cell layers of the retina. In mouse, retinal ganglion cell axons expressing this gene projected to the suprachiasmatic nucleus and other brain nuclei involved in circadian photoentrainment. In mouse, this protein is coupled to a transient receptor potential ion channel through a G protein signaling pathway and produces a physiologic light response via membrane depolarization and increased intracellular calcium. The protein functions as a sensory photopigment and may also have photoisomerase activity. Experiments with knockout mice indicate that this gene attenuates, but does not abolish, photoentrainment.

Supplementary Table S2: The circadian genes, SNPs, base change, MAF/GMAF, Hardy-Weinberg values and genotyping rate

Gene	Pathway	SNP db rs #	Position/SNP type	Base or AA change	MAF/GMAF	H-W eq. (X^2, *P* value)	Genotyping rate (controls/cases)
ARNTL1/BMAL1	Core circadian	rs7950226	11p15/tagging	G/A	0.46/0.44	0.97, 0.32	96.1/93.4
ARNTL1/BMAL1	Core circadian	rs2290035	11p15/tagging	T/A	0.43/0.40	0.09, 0.76	94.1/95
ARNTL1/BMAL1	Core circadian	rs2278749	11p15/tagging	C/T	0.19/0.15	4.73, 0.03	92.3/93.9
ARNTL1/BMAL1	Core circadian	rs7126303	11p15/tagging	T/C	0.44/0.41	0.001, 0.99	92.1/92.8
ARNTL1/BMAL1	Core circadian	rs1481892	11p15 tagging	C/G	0.29/0.31	0.02, 0.88	89.5/87.3
ARNTL1/BMAL1	Core circadian	rs969485	11p15/ tagging	A/G	0.25/0.40	0.04, 0.83	89.7/89.5
ARNTL2/BMAL2	Core circadian	rs2306074	12p12/tagging	T/C	0.34/0.41	0.04, 0.83	97/97.3
ARNTL2/BMAL2	Core circadian	rs4964052	12p12/tagging	G/T	0.43/0.40	0.18, 0.67	96.1/97.3
ARNTL2/BMAL2	Core circadian	rs35670208	12p12 AA change Asp/Glu	T/G	ND/ND	ND	93.3/93.7
ARNTL2/BMAL2	Core circadian	rs4964059	12p12/tagging	A/C	0.36/0.29	2.22, 0.13	90.9/91.4
CLOCK	Core circadian	rs17776421	4q12/tagging	G/A	0.36/0.43	0.12, 0.72	97.1/96.8
CLOCK	Core circadian	rs1048004	4q12/tagging	C/A	0.26/0.22	0.51, 0.47	96.8/97.5
CLOCK	Core circadian	rs11133376	4q12/tagging	T/C	0.35/0.29	0.14, 0.70	96.5/97.7

CLOCK	Core circadian	rs3749474	4q12/tagging	C/T	0.40/0.39	4,59, 0.032	96.1/96.2
CLOCK	Core circadian	rs1801260	4q12/tagging	A/G	0.26/0.22	0.83, 0.36	95.3/95.3
CLOCK	Core circadian	rs11133373	4q12/tagging	C/G	0.36/0.34	0.019, 0.89	95.3/94.1
CLOCK	Core circadian	rs13102385	4q12/tagging	C/T	0.35/0.40	0.38, 0.54	91.7/89.1
CLOCK	Core circadian	rs7698022	4q12/tagging	A/C	0.26/0.22	0.26, 0.63	90.7/90.1
CRY1	Core circadian	rs3809235	12q23-24.1/5'UTR	T/C	0.45/0.45	0.31, 0.51	96.1/95.2
CRY1	Core circadian	rs12315175	12q23-24.1/tagging	T/C	0.16/0.23	4.84, 0.03	90.0/91.8
CRY2	Core circadian	rs11605924	11p11.2/tagging	A/C	0.48/0.33	0.50, 0.48	97.5/98
CRY2	Core circadian	rs2292912	11p11.2/tagging	G/C	0.24/0.49	0.15, 0.64	96.5/97.5
CRY2	Core circadian	rs7123390	11p11.2/tagging	G/A	0.27/0.17	1.32, 0.25	96.5/97.1
CRY2	Core circadian	rs12364060	11p11.2/tagging	C/T	0.22/0.13	7.01, 0.008	91.9/89.1
CRY2	Core circadian	rs11038689	11p11.2/tagging	A/G	0.25/0.15	5.96, 0.014	88.2/87.1
CRY2	Core circadian	rs1401417	11p11.2/tagging	C/G	0.24/0.15	2.95, 0.08	84.5/80.1
CRY2	Core circadian	rs10838524	11p11.2/tagging	G/A	0.48/0.35	0.50, 0.48	93.7/91.7
CRY2	Core circadian	rs12281674	11p11.2/tagging	A/G	0.05/0.10	0.036, 0.85	91.1/92.2
CSNK1E	Core circadian	rs1534891	22q13.1/CGEMS	G/A	0.13/0.10	0.19, 0.66	96.1/94.6

CSNK1E	Core circadian	rs5757037	22q13.1/tagging	C/T	0.35/0.37	0.22, 0.64	94.8/93.4
NPAS2	Core circadian	rs2305160	2q11.2/AA change	G/A, Ala394Thr	0.37/0.23	0.87, 0.35	97/97.1
NPAS2	Core circadian	rs1369481	2q11.2/tagging	C/T	0.29/0.24	0.60, 0.43	97/96.2
NPAS2	Core circadian	rs17024926	2q11.2/tagging	T/C	0.35/0.37	1.52, 0.22	97/94.6
NPAS2	Core circadian	rs7565018	2q11.2/tagging	A/G	0.24/0.47	0.11, 0.73	95.8/92.8
NPAS2	Core circadian	rs4074920	2q11.2/tagging	A/G	0.23/0.30	0.02, 0.88	81.5/83.2
PER1	Core circadian	rs885747	17p13.1-p12/tagging	C/G	0.43/0.35	0.35, 0.55	97.1/97.3
PER1	Core circadian	rs2253820	17p13.1-p12/tagging	G/A	0.14/0.14	0.54, 0.46	93.8/91.8
PER1	Core circadian	rs2289591	17p13.1-p12/tagging	G/T	0.25/0.12	0.24, 0.62	89.9/91
PER2	Core circadian	rs11695472	2q37.3/tagging	A/C	0.26/0.16	0.77, 0.36	96.6/96.2
PER2	Core circadian	rs7602358	2q37.3/tagging	T/G	0.19/0.16	0.45, 0.50	87.9/88.2
PER3	Core circadian	rs228697	1p36.23/AA change	C/G	0.10/0.06	3.02, 0.08	96.5/95.2
PER3	Core circadian	rs1012477	1p36.23/tagging	G/C	0.17/0.13	2.55, 0.11	95.8/93
PER3	Core circadian	rs10462020	1p36.23/AA change (Val/Gly)	T/G	0.19/0.12	0.25, 0.61	93.4/93/4
ROR-a	Core circadian	rs11630262	15q22/tagging	C/T	0.16/0.12	0.056, 0.81	95.8/95

ROR-a	Core circadian	rs3743266	15q22/tagging	A/G	0.28/0.29	0.47, 0.49	95.6/94.1
ROR-a	Core circadian	rs10438343	15q22/tagging	G/A	0.19/0.17	0.68, 0.41	92.2/91.9
ROR-a	Core circadian	rs7165874	15q22/tagging	A/T	0.39/0.39	0.12, 0.72	91.9/90.7
ROR-b	Core circadian	rs3903529	9q22/tagging	T/A	0.29/0.18	0.06, 0.80	95.1/94.1
ROR-b	Core circadian	rs7022435	9q22/tagging	G/A	0.25/0.16	0.65, 0.42	93.1/94.8
ROR-b	Core circadian	rs3750420	9q22/tagging	C/T	0.31/0.36	1.09, 0.29	92.7/93
TIMELESS	Core circadian	rs774047	12q12-13/AA change	G/T	0.43/0.49	0.007, 0.99	97/95.9
TIMELESS	Core circadian	rs774027	12q12/AA change	A/T	0.43/0.49	0.056, 0.81	94.4/94.6
TIMELESS	Core circadian	rs2291739	12q12/AA change	T/C	0.39/0.43	5.19, 0.022	87/86.4
AANAT	Melatonin synthesis	rs3760138	17q25/tagging	T/G	0.44/0.40	2.69, 0.13	97.3/97
AANAT	Melatonin synthesis	rs28936679	17q25/AA change/Ala/Thr	G/T	0.000/0.008	ND	97.1/96.2
AANAT	Melatonin synthesis	rs4238989	17q25/tagging	G/C	0.42/0.43	0.02, 0.88	96.1/96.4
MTNR1A	Melatonin receptor 1	rs2119882	4q35/tagging	G/A	0.41/0.49	8.42, 0.003	95.3/95.7

MTNR1A	Melatonin receptor 1	rs13113549	4q35/tagging	T/C	0.44/0.46	4.72, 0.03	94.8/96.2
MTNR1B	Melatonin receptor 2	rs10830963	11q21/tagging	C/G	0.24/0.26	1.04, 0.31	92.9/93.7
OPN4/MOP	Melatonin synthesis	rs2675703	10q21/AA change/Pro/Leu	C/T	0.11/0.10	2.73, 0.10	91.4/89.1

MAF, minor allele frequency in controls in this study population compared to **GMAF (G**lobal Minor Allele Frequency) from 1000 genome study as reported by NCBI's SNP database (http://www.ncbi.nlm.nih.gov/SNP/ftpfile.cgi?FileID=120330073353); **CGEMS,** Cancer Genetic Marker of Susceptibility; **ND**, not determined.
